# New Genomic Approach Predicts True Evolutionary History of Bacterial Genomes

**DOI:** 10.1371/journal.pbio.0000031

**Published:** 2003-09-15

Bacteria are an indiscriminate lot. While most organisms tend to pass their genes on to the next generation of their species, bacteria often exchange genetic material with totally unrelated species. That is why skeptics doubted that bacteria researchers could ever hope to map a reliable history of cell lineages in bacteria over time. But now, thanks to the availability of sequenced genomes for groups of related bacteria, researchers at the University of Arizona demonstrate that constructing a bacterial family tree is indeed possible.

Previous efforts to trace the ancestry of bacteria were constrained by a dearth of related bacterial genomes, which, among other things, prevented scientists from successfully accounting for bacteria's tendency to exchange genes with unrelated organisms. In this process, called *lateral gene transfer*, organisms acquire genetic material not from their ancestors, the most prevalent route, but from unrelated organisms. Lateral gene transfer greatly complicates the issue of who descended from whom, because two organisms could appear closely related based on the similarity of some genes but distantly related based on other genes. The problem is to determine which genes have been faithfully vertically transmitted—from parent cell to offspring—and thus reflect the history of the bacterial cell lineages.

In this issue, Nancy Moran, Emmanuelle Lerat, and Vincent Daubin propose an approach that solves this problem by identifying a set of genes that serve as reliable indicators of the vertical transfer of bacterial cell lineages. This method has important implications for biologists studying the evolutionary history of organisms by establishing a foundation for charting the evolutionary events, such as lateral gene transfer, that shape the structure and substance of genomes. With this method, scientists can begin to understand how bacteria have evolved and how their genomes have changed.

Bacteria promise to reveal the most information about genomic evolution, because so many clusters of related bacterial genomes have been sequenced—allowing for broad comparative analysis among species—and their genomes are small and relatively compact. In this study, the researchers chose the ancient bacteria group Proteobacteria, an ecologically diverse group (including Escherichia coli and Salmonella species) with the most documented cases of lateral gene transfer and the highest number of species with sequenced genomes.

The researchers identified a set of likely single-copy orthologs (homologous genes that diverged due to the speciation of ancestral lineages) with widespread distribution in the different species of Proteobacteria that could be used to trace the history of the cell lineages. Surprisingly, they found that almost all of the 205 ortholog gene families they selected supported the same evolutionary branching pattern. Only two did not, which the researchers then investigated and found to be the result of lateral gene transfer.

These results, the researchers say, support the ability of their method to reconstruct the important evolutionary events affecting genomes. By mapping out the evolutionary path of genetic information on a genomic level, their approach promises to elucidate not only the evolution of bacterial genomes but also the diversification of species.

**Figure:**
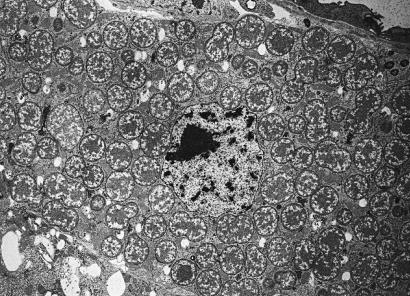
Electron micrograph of Proteobacteria in eukaryotic cell

